# Improvements in hip stability and knee muscle strength after a single-dose platelet-rich plasma injection combined with exercise in women with knee osteoarthritis

**DOI:** 10.1007/s11845-026-04390-3

**Published:** 2026-04-21

**Authors:** Muharrem Gökhan Beydagi, Sibel Bozgeyik Bağdatli, Yusuf Topal, Gizem Irem Kinikli, Gazi Huri, Hande Güney-Deniz

**Affiliations:** 1 Faculty of Health Sciences, Department of Physiotherapy and Rehabilitation, Firat University, Elazığ, Turkey; 2 Faculty of Physical Therapy and Rehabilitation, Department of Musculoskeletal Physiotherapy and Rehabilitation, Hacettepe University, Ankara, Turkey; 3 Faculty of Health Sciences, Department of Physiotherapy and Rehabilitation, Kırşehir Ahi Evran University, Kırşehir, Turkey; 4 Faculty of Medicine, Department of Orthopedics and Traumatology, Hacettepe University, Ankara, Turkey

**Keywords:** Knee osteoarthritis, Platelet-rich plasma, Exercise therapy, Hip muscle strength, Hand-held dynamometer, Pain

## Abstract

**Background:**

Knee osteoarthritis (OA) is associated with pain and lower-limb muscle weakness. Platelet-rich plasma (PRP) may reduce symptoms, but functional recovery may depend on concurrent exercise. Aims: To evaluate changes in pain and knee/hip muscle strength after a single intra-articular PRP injection combined with a 6-week home exercise programme in women with knee OA.

**Methods:**

In this single-group longitudinal study, 27 women (mean age 57.5±7.3 years) performed home exercises three times weekly for 6 weeks after unilateral PRP injection. Outcomes were assessed at baseline (5 days post-injection), week 6, and week 12: hip stability isometric strength using the Hip Stability Isometric Test (HipSIT), quadriceps and hamstring strength (hand-held dynamometry; N/kg), and activity pain using a visual analog scale (VAS, 0–10). Repeated-measures ANOVA was used.

**Results:**

On the affected side, The Hip Stability Isometric Test (HipSIT) increased from 2.68±1.19 to 3.15±1.13 at week 6 and 3.25±1.19 at week 12 (p=0.006). Quadriceps strength increased from 2.57±0.91 to 2.95±0.98 and 2.88±1.07 (p=0.001), and hamstring strength from 1.43±0.56 to 1.67±0.59 and 1.69±0.56 (p=0.011). Activity pain decreased from 7.27±2.10 to 4.48±2.42 and 4.22±2.93 (p=0.001).

**Conclusions:**

A single PRP injection followed by a short home exercise programme was associated with reduced pain and improved hip stability and knee muscle strength up to 12 weeks in women with knee OA.

## Introduction

The available non-surgical treatments for knee osteoarthritis (OA) include both pharmacological and non-pharmacological options. Clinical guidelines for managing knee OA recommend a core set of non-pharmacological interventions, including patient education, weight loss (for those who are overweight), and structured strengthening exercises for lower extremity muscles [1, 2]. Currently, pharmacological treatments such as Platelet-Rich Plasma (PRP) injections have been extensively investigated for treating knee OA [3, 4]. Several trials have shown that PRP might be a promising alternative in reducing disease related symptoms [5, 6]. It has been demonstrated that intra-articular PRP injections can lead to improvements above the minimal clinically important difference in terms of pain and function [7].

PRP injections provide significant benefits in pain and function improvements in the treatment of knee OA. In addition, the available literature suggests that PRP shows a significant increase in pain reduction and function improvement when used in combination with exercise therapy. A combination of PRP and structured exercise programs is recommended to enhance lower limb muscle strength recovery and relieve pain in patients with knee OA [4, 8, 9]. A systematic review and meta-analysis of randomized controlled trials found that PRP injections combined with exercise therapy showed better outcomes in terms of pain reduction and functional improvement compared to PRP or exercise therapy alone [10]. Exercises aimed at strengthening the muscles around the hip and knee enhance the healing effects of PRP and improve overall treatment outcomes [11, 12].

Weakness in the lower extremity muscle group, particularly the knee extensor muscle, is closely associated with the development and progression of joint degeneration in patients with knee OA [13, 14]. In fact, muscle weakness may precede the onset of the disease and play a role in its pathophysiology [15]. Although studies on knee OA have generally focused on the importance of quadriceps strength, there has been comparatively little emphasis on the significance of hip muscle group strength. Weakness of the muscles around the hip may increase the load on the knee joint. In particular, weakness of the gluteus medius and minimus muscles can reduce hip stability, which can lead to increased load on the knee joint. This may lead to an imbalance of the mechanical load on the knee joint, accelerating the progression of knee OA [16]. Chang et al. reported that a greater hip abduction moment may prevent excessive loading of joint and protected against the progression of knee OA [17]. Also, the gluteus maximus muscle holds the knee joint in extension by placing the line of gravity posteriorly. Weakness in this muscle increases the demand on the knee musculature for the knee extension position, thus increasing the muscular compressive forces on the knee joint [18]. In this light, it is understood that the hip stability generated by the muscles around the hip joint [especially hip extensor, abductor and external rotator (ER)] has an important place in the treatment of knee OA. When the literature is reviewed, the effect of the treatments given in knee OA on the recovery of the hip muscles is limited.

Herewith, there is limited trial in the literature that deals with the effect of a combination of PRP injection with strengthening exercises on lower limb muscles strength (especially hip muscle function) recovery and pain in patients with knee OA. The aim of the present study was to investigate the effects of a combination of PRP injection with strengthening exercises for lower extremity muscles on pain, knee and hip muscle function (quadriceps, hamstrings and hip extensor, abductor and external rotator) recovery in patients with knee OA. We hypothesized that a combination of PRP injection with strengthening exercises for lower extremity muscles would decrease pain severity and improve knee and hip muscle function in knee OA.

## Methods

### Study design

A longitudinal study design using a single-group repeated measures approach was utilized to assess knee and functional hip strength in patients with knee OA at pre-treatment, post-treatment (6-week), and 12-week follow-up intervals. Approval for the study protocol was obtained from the University Ethics Committee (decision no: FTREK24/110 ,date: 10/07/2025), and all procedures adhered to the principles outlined in the Declaration of Helsinki. Prior to enrollment, all participants provided informed consent by reading and signing a consent form.

### Sample size

We conducted a post hoc power analysis according to The Hip Stability Isometric Test - HipSIT recovery using (G*power; v3.1.9.2, Heinrich-Heine-University, Dusseldorf, Germany). Based on our sample size of 27 and an alpha level of 0.05, the study had > 95% power with an effect size of 0.52. Although a post hoc power analysis indicated that the study had sufficient statistical power (> 95%), an a priori sample size calculation was not performed. Therefore, the findings of this study should be interpreted with some caution in terms of methodological rigor.

### Participants

This study was conducted on female patients with knee OA who were referred by a single orthopedic surgeon to the physiotherapy department following a unilateral single-dose PRP injection.

The inclusion criteria were aged between 45 and 70 year, diagnosed with grade II and III KOA for affected knee and grade II or less KOA for unaffected knee according to Kellgren-Lawrence osteoarthritis classification and a history of unilateral knee pain during activity with a VAS score of at least 5 in the past 1–3 months. The exclusion criteria consisted of previous any intra-articular injections to the knee within the past 6 months, performing regular strengthening exercise for lower extremities or receiving non-pharmacological treatment for knee OA within the past 6 weeks, history of any lower extremity surgery, history of any neurological, rheumatological and/or cognitive diseases, unable to use a telephone.

Baseline assessments were conducted 5 days following intraarticular PRP injection (pre-exercise; 5 days after PRP). This time point was selected to minimize the potential influence of acute post-injection reactions and to allow stabilization of symptoms before baseline measurements. Twenty-eight eligible patients were scheduled according to inclusion criteria. All 27 patients completed the 12-week follow-up.

### Treatment

#### Platelet-rich plasma (PRP) injection (PRP)

The PRP injection was administered intra-articularly to the affected knee by an experienced orthopedic surgeon under sterile conditions in a hospital setting. The patient was positioned in the supine position with the knee in slight flexion to facilitate joint access. Following standard skin antisepsis, the injection was performed using anatomical landmarks via a conventional approach. Ultrasound guidance was not used.

PRP was prepared using a standard centrifugation protocol in accordance with routine clinical practice at the institution. The preparation aimed to obtain autologous platelet-rich plasma suitable for intra-articular administration. However, detailed parameters regarding centrifugation settings, platelet concentration, injected volume, and leukocyte content (leukocyte-rich vs. leukocyte-poor PRP) were not recorded in the scope of this study.

#### Exercise programme

Following baseline measurements a brief education about the disease and its risk factors was provided. The patients were were instructed to perform the exercises three times per week for a period of 6-week at home. The exercise programme is primarily designed to strengthen the knee extensor, flexor and hip extensor, abductor and external rotator muscles (Appendix 1). The same physiotherapist also educated the participants of the benefits of increased levels of physical activity, and they were encouraged to walk regularly at least 30 min per day, five days per week. Exercise intensity was progressively adjusted throughout the 6-week period based on patient tolerance and performance. Adherence to the exercise program was monitored through weekly phone calls and patient self-reports.

All measurements were applied at baseline (pre-exercise; 5 days after PRP), at 6th and 12th weeks following exercise application.

### Outcome measurements

#### Muscle-strength testing

The primary outcome was HipSIT (the maximum isometric muscle strength of hip extensor, abductor and external rotator) using a hand-held dynamometer (Lafayette Instrument Company, Lafayette, IN, USA). The HipSIT is a test that provides a more three-dimensional evaluation opportunity by allowing the strength of hip muscle groups to be more functional than measuring these muscles individually [19]. With this measurement method, hip extensor, abductor and external rotator can be evaluated together. For this measurement, the subjects were asked to lie on their side in the clam position (hips at 45°, knees at 90° flexion, heels in contact) with the lower extremity to be evaluated on top. The hip was abducted to ER without breaking the contact of the heels. The hand dynamometer was placed 5 cm above the lateral knee and the measurement was performed. The region was fixed with an inelastic band to prevent any different movement in the hip region.

The maximum isometric muscle strength of quadriceps femoris and hamstrings muscles also measured by a hand-held dynamometer (Lafayette Instrument Company, Lafayette, IN, USA). Quadriceps femoris muscle strength was measured in the sitting position (hip in 90° and knee in semi-flexion position) with the hands crossed over the chest. The hand dynamometer was placed on the anterior surface of the ankle during the test [20]. Hamstring muscle group strength measurement was performed in prone position (hip neutral position, knee flexion 90°). The hand dynamometer was placed on the proximal posterior surface of the ankle [21].

Before measurement each patient was verbally informed to ensure correct movement and subjects were asked to perform a single submaximal trial against the evaluator’s hand to familiarize participants with the procedure. A total of 3 trials of maximal effort (each of 5 s duration) were performed. 2 min rest was given between the trials. There was also a 5-minute rest interval between muscle groups measurements. The maximum force output (N) from the 3 trials was normalized to express strength relative to body mass (N/kg) [22].

The index of HipSIT, quadriceps and hamstring strength were calculated were used to define strength symmetry (isometric muscle strength of affected side/ isometric muscle strength of unaffected side*100) [23].

#### Pain

The secondary outcome was knee pain severity during activity was evaluated using Visual analog scale (VAS). The VAS was assessed using numbers ranging from 0 to 10, spaced 10 cm apart. Patients were asked to indicate the appropriate score on the line indicating their personal pain during activity (0 = no pain, 10 = worst imaginable pain) [24].

All muscle strength measurements were evaluated bilaterally by the same physiotherapist.

### Statistical analyses

Data analyses were performed using IBM SPSS Statistics version 23.0 (IBM Corp., Armonk, NY, USA). Normality of continuous variables was assessed using the Shapiro–Wilk test. Descriptive data were presented as mean ± standard deviation. Changes over time [baseline (pre-exercise; 5 days after PRP), week 6, and week 12] in HipSIT strength, quadriceps and hamstring isometric strength (N/kg), and activity-related pain (VAS) were analyzed using one-way repeated-measures ANOVA. Sphericity was assessed with Mauchly’s test; when violated, the Greenhouse–Geisser correction was applied. When the overall test was significant, post-hoc pairwise comparisons between time points were performed with Bonferroni adjustment. A two-sided p value < 0.05 was considered statistically significant.

## Results

Demographic characteristics of participants were presented in Table 1.

**Table 1:** Descriptive parameters for the patients

Parameters (*n* = 27)		Mean ± SD (min-max)
Age (year)		57.48 ± 7.29 (45–70)
Height (cm)		158.33 ± 4.26 (151–167)
Weight (kg)		72.03 ± 15.99 (45–106)
BMI (kg/m^2^)		28.76 ± 6.33 (19.74–43.70)
Pain Duration (year)		5.16 ± 5.20 (0.16-20)
Affected side (R/L)		15/12
KL grade	**2**	12(%42,8)
	**3**	15(%57,2)

*BMI* body mass index, *KL* *Kellgren*-*Lawrence; SD* Standard deviation, *R* Right, *L* Left

Significant time effect was observed in HipSIT (F_1.4,35.7_=7.125, *p* = 0.006), in quadriceps muscle strength (F_2,52_ =9.94, *p* ≤0.001), in hamstring muscle strength (F_1.5,40.1_=5.691, p **=** 0.011) and for affected side (Table 2). HipSIT improved from baseline to 6th week (mean difference = 0.46; 95% CI = 0.03–0.90.; *p* = 0.033) and from baseline to 12th week (mean difference = 0.56; 95% CI = 0.06-1.08-; *p* = 0.024). Quadriceps muscle strength improved from baseline to 6th week (mean difference = 0.37; 95% CI = 0.12–0.62; *p* = 0.002) and from baseline to 12th week (mean difference = 0.30; 95% CI = 0.05–0.54; *p* = 0.011). Hamstring muscle strength improved from baseline to 12th week (mean difference = 0.26; 95% CI = 0.02–0.49; *p* = 0.027).

**Table 2:** Outcome measures for affected and unaffected knees at baseline, 6th week after home exercise and 12th week follow-up

		Baseline (*n* = 27)		6th week (post-treatment) (*n* = 27)		12th week (follow-up) (*n* = 27)		*P*	Effect size	*F*
		Mean±SD	95%CI	Mean±SD	95%CI	Mean±SD	95%CI	≤0.001*	0.277	(2,52) 9.94
Quadriceps strength (N/kg)	Affected knee	2.57±0.91	2.21–2.94	2.95 ±0.98	2.56–3.34	2.88±1.07	2.45–3.30			
	Unaffected knee	2.82±0.96	2.43–3.20	2.85±1.01	2.45–3.25	2.93±1.00	2.54–3.31	0.417	0.028	(1.2,31.1) 0.748
Hamstring strength (N/kg)	Affected knee	1.43±0.56	1.21–1.65	1.67±0.59	1.43–1.90	1.69±0.56	1.47–1.92	0.011*	0.180	(1.5,40.1) 5.691
	Unaffected knee	1.67±0.69	1.39–1.95	1.77±0.61	1.53–2.02	1.72±0.53	1.50–1.93	0.472	0.021	(1.1,28.5) 0.568
Hip stability isometric strength (N/kg)	Affected knee	2.68±1.19	2.20–3.15	3.15 ±1.13	2.70–3.60	3.25±1.19	2.78–3.72	0.006*	0.215	(1.4,35.7) 7.125
	Unaffected knee	2.98±1.23	2.49–3.46	3.26±1.25	2.76–3.75	3.22±1.24	2.73–3.71	0.085	0.098	(1.5,38.9) 2.834
QI (%)		92.30 ± 14.78	86.48–98.12	103.92 ± 17.12	97.16–110.70	97.01 ± 15.50	90.87-103.14	**0.004**	0.190	(2,52) 6.119
HI (%)		89.91 ± 2.97	83.79–96.03	94.35 ± 2.62	88.95–99.74	98.61 ± 3.37	91.66-105.57	0.070	0.097	(2,52) 2.802
HipI (%)		89.95 ± 3.58	82.59–97.31	97.35 ± 2.48	92.25-102.45	102.03 ± 3.39	95.06-109.01	**0.008**	0.190	(15.6,40.6) 6.080
Pain level (cm)		7.27±2.10	6.43–8.10	4.48±2.42	3.52–5.43	4.22±2.93	1.90–6.30	≤0.001*	0.403	(1.6,40.9) 17.551

*P < 0.05, Repeated-measures ANOVA; *QI* Quadriceps Index, *HI* Hamstring Index, *HipI* Hip Stability Isometric Index

Significant time effects were observed for Hip symmetry index (HipI) (F_15.6,40.6_=6.080, p **=** 0.008) and Quadriceps Index (QI) (F_2,52_ =6.119, p **=** 0.004). No significant time effect was observed in Hamstring Index (HI) (*p* = 0.070). HipI increased from baseline to 12th week (mean difference = 12.08; 95% CI = 1.16–22.99; *p* = 0.026) and QI increased from baseline to 6th week (mean difference = 11.59; 95% CI = 2.59–20.60; *p* = 0.009). There was no difference in HI between any time points (*p* > 0.05).

There were no differences in hamstring strength from baseline to 6th week (*p* = 0.076) and from 6th week to 12th week (*p* = 1.000). Additionally, no significant differences were observed in HipSIT (*p* = 0.889) from 6th week to 12th week and in quadriceps strength (*p* = 0.100).

No significant time effects observed HipSIT (*p* = 0.085), in quadriceps strength (*p* = 0.417) and hamstring strength (*p* = 0.472) on the unaffected side (Table 2).

Significant time effect was observed for pain severity (_F1.6,40.9_=17.551, *p* = 0.001). The pain severity significantly decreased from baseline to 6th week (mean difference = 2.79; 95% CI = 1.67–3.90; p**≤**0.001) and from baseline to 12th week (mean difference = 3.05; 95% CI = 1.27–4.83; p**≤**0.001). However, there was no significant difference in pain severity from 6th week to 12th week (*p* = 1.000).

## Discussion

The study demonstrated that a single-dose intra-articular PRP injection combined with an exercise program was associated with reductions in pain and improvements in muscle strength (HipSIT, quadriceps and hamstrings) in patients with knee OA. The findings indicated that the strengthening exercises were effective for up to 12 weeks following the PRP injection. Our results showed that muscle strength began to increase within the first six weeks, while pain reduction was observed at both the sixth and twelfth weeks.

PRP aims to stimulate cartilage repair, alleviate OA symptoms, and delay the need for joint replacement surgery in patients with knee OA [25, 26]. McLarnon et al., indicated that intra-articular PRP significantly reduced symptoms in individuals with knee OA when compared to placebo injection [27]. Additionally, exercise programs are an essential treatment for knee OA as they reduce pain and increase muscle strength [28]. Clinical practice guidelines recommend programs that specifically include lower limb muscle strengthening exercises as a crucial component of knee OA management [1, 29]. Çakmak et al., reported that the exercise therapy following PRP is more effective than PRP alone in reducing pain and increasing muscle strength [8]. Badr et al. found that exercise therapy after PRP applications yielded better results in improving pain compared to PRP alone or an exercise program alone [30]. Similarly, Bozgeyik et al., emphasised that the exercise programme applied after PRP in individuals with knee OA is important in reducing pain, improving muscle strength and improving function [9]. Similarly, our findings indicate that intra-articular PRP injection combined with an exercise program can reduce pain and improve muscle strength (HipSIT, quadriceps and hamstrings) in patients with knee OA.

Muscle weakness is both a cause and a result of knee OA. One of the most important indicators of pain and disability in patients with OA is the loss of muscle strength. For this reason, treatment strategies, applied to reduce pain and disability, mostly focus on increasing muscle strength [31]. PRP application has been shown to reduce pain, stiffness, and disability in patients with knee OA. In addition, it is recommended that additional exercise programs be applied to ensure muscle strength recovery [4]. Soylu et al., found that the exercise program applied together with PRP in patients with knee OA had a significant effect on the muscle strength of the patient [32]. In a study conducted by Yung-Tsan Wu et al. in patients with knee OA, it was reported that no significant increase in muscle strength was obtained after PRP injection. They stated that the reason for this was the lack of exercise programme especially after PRP injection [4]. Similarly, our study demonstrated that muscle strength (quadriceps, hamstring, and hip muscle) improved after platelet-rich plasma injection in combination with a home exercises program. In our study, muscle evaluation was repeated at the 6th and 12th weeks following the treatment. A significant increase (quadriceps, hamstring, and hip muscle) was observed at 6th week, but no significant increase was observed at 12th weeks from 6th week. This may be explained by the fact that the exercise program was applied for only 6 weeks.

Muscle weakness in the development and progression of knee osteoarthritis is not limited to the quadriceps and hamstring muscles but also includes the muscles around the hip [33]. Hip muscle weakness has been observed in people with knee OA and it is suggested that this increases load on the knee joint [34]. Considering the strength deficits in the hip muscles, a targeted exercise program for hip muscle strengthening might reduce knee loading and improve knee symptoms. However, the number of studies on the strengthening of hip muscles was less than that of quadriceps and hamstring. The recent a systematic review highlighted that in conservative treatment of knee osteoarthritis, hip muscle strengthening alone or in combination with lower extremity exercises demonstrated improvement in symptoms without any significant effect on knee loading. The same study recommended a series of exercise interventions focused on strengthening the lower limb muscles in the treatment of people with knee OA [35]. However, less is known about the the effect of exercise programme, includes the muscles of around the hips strengthening, applied after PRP in knee OA on the recovery of the hip muscles. The results of our study showed that after a single dose of PRP injection, exercise therapy aimed at increasing the strength of the muscles around the hip and knee improved hip muscle strength. The hip stability generated by the muscles around the hip joint (especially hip extensor, abductor and external rotator) has an important place in the treatment of knee OA in terms of loads on the knee. It may be explained by the inclusion of the muscles around the hip in the exercise therapy given after PRP will be very important in reducing the symptoms of the patients and preventing the progression of knee osteoarthritis.

Also consistent with previous studies, our study demonstrated that pain reduced after PRP injection in combination with a home exercises program. The magnitude of pain reduction observed in this study exceeds the minimal clinically important difference (MCID) reported for VAS in patients with knee osteoarthritis (approximately 1.5–2 points), indicating that the improvement is clinically meaningful. A possible explanation for this reduction in pain could be the synergistic effects of PRP injection and the exercise program. PRP injection may contribute to this effect through the immediate and sustained release of growth factors, which can enhance healing and promote tibiofemoral cartilage regeneration in the knee, resulting in sustained clinical benefits [36]. Furthermore, exercises in individuals with knee OA have antinociceptive effects and general health benefits [37]. The results of our study demonstrated that the exercise program following PRP injection enhanced the therapeutic effect on pain symptoms. In our study, it may be explained by strengthening the quadriceps and hamistring muscles as well as strengthening the muscles around the hip increased the exercise-induced hypoalgesia. This combined treatment—PRP injection followed by an exercise program—proved more effective in improving pain symptoms, and we recommend implementing our exercise program following PRP injection.

Consequently, it is important to examine the effects of treatments applied in knee OA not only on the strength development of the muscles around the knee, but also on the strength development of the muscles around the hip. To our knowledge, this was the first study that evaluated hip muscle strength after single dose PRP injections in combination with a home exercises program. In our study, hip muscle evaluation was repeated at the 6th and 12th weeks following the treatment. Our study demonstrated that hip muscle strength increased at the 6th week from baseline. The increase in muscle strength may have been due to a reduction in the pain symptom, making patients more willing to use the knee joint. The reduction in the pain symptom seen with the effect of PRP also provides control of arthrogenic muscle inhibition. The combination of PRP with exercise will increase muscle strength and provide better improvements.

### Our study strengths

When the literature on knee OA is examined, we think that this is the first study to examine the effects of this treatment strategy (home exercises program after a single dose PRP injections) on pain and muscle strength (especially on the muscles around the hip). In this sense, we think that it will make important contributions to the literature.

### Limitations

Among the limitations of our study are that we applied only 6 weeks of exercise and we did not evaluate the long-term results after single PRP injections in combination with a home exercises program. Another limitation of the study is that muscle strength was evaluated using a hand-held dynamometer. The muscle strength of patients with OA should be evaluated with isokinetic devices to be more objective. However, in the literature, hand dynamometer commonly used in evaluating muscle strength in knee [38, 39]. Another limitation is that only female patients were included in the study; therefore, the findings may not be generalizable to male patients with knee osteoarthritis. In addition, an a priori sample size calculation was not performed, which may limit the methodological robustness of the study despite the high post hoc power.

## Conclusion

In conclusion, there was an improvement in pain and lower extremity muscle strength after single dose PRP injections in combination with a home exercises program in patients with knee OA. Results obtained from this study suggest that the regimen (single PRP injection in combination with a home exercise program) may be associated with reductions in pain and improvements in knee and hip muscle strength in the short term (12-week follow-up). For this reason, we recommend that exercise programs be included in recovery programs, even if only a single dose of PRP injection is given in these patients.

## Data Availability

The datasets used and/or analysed during the current study are available from the corresponding author on reasonable request.

## Appendix. Home exercises program and description

**Table:**

	Description
Quadriceps Strengthen Exercises	-Isometric contraction in supine with 5 s hold (10–15 repetition) -Seated knee extension with 90^o^ hip flexion with 5 s hold (10–15 repetition) -Straight Leg Raise in supine with 5 s hold (10–15 repetition)
Hamstring Strengthen Exercise	Isometric contraction in supine with 5 s hold (10–15 repetition) Active knee flexion in prone with 5 s hold (10–15 repetition)
Hip Stabilator Strengthen Exercises	Side-lying bent-leg hip abduction (clams) with 5 s hold (10–15 repetition) Side leg raise hip abduction with 5 s hold (10–15 repetition)
Steps Exercises	Step-ups (affected leg on the step, control knee straightening, lower to start position by controlling knee bending) (10–15 repetition) Forward touchdowns from a step (affected leg on the step, control knee bending to lightly tap floor in front with toes of non-affected leg, return to start by controlling knee straightening) (10–15 repetition) Side stepping (10–15 repetition)
Sit-to-stand Exercises	Sit to stand from a standard height chair without using hands/arms (10–15 repetition)
Squat Exercises	Partial wall squats with weight distributed bilaterally (10–15 repetition)
